# Searching for the Mechanisms of Mammalian Cellular Aging Through Underlying Gene Regulatory Networks

**DOI:** 10.3389/fgene.2020.00593

**Published:** 2020-06-30

**Authors:** Wenbo Li, Lei Zhao, Jin Wang

**Affiliations:** ^1^State Key Laboratory of Electroanalytical Chemistry, Changchun Institute of Applied Chemistry, Chinese Academy of Sciences, Changchun, China; ^2^Department of Chemistry and Physics, State University of New York at Stony Brook, Stony Brook, NY, United States

**Keywords:** aging, slow-aging, landscape, flux, entropy production, gene regulatory network

## Abstract

Aging attracts the attention throughout the history of humankind. However, it is still challenging to understand how the internal driving forces, for example, the fundamental building blocks of life, such as genes and proteins, as well as the environments work together to determine longevity in mammals. In this study, we built a gene regulatory network for mammalian cellular aging based on the experimental literature and quantify its underlying driving force for the dynamics as potential and flux landscape. We found three steady-state attractors: a fast-aging state attractor, slow-aging state attractor, and intermediate state attractor. The system can switch from one state attractor to another driven by the intrinsic or external forces through the genetics and the environment. We identified the dominant path from the slow-aging state directly to the fast-aging state. We also identified the dominant path from slow-aging to fast-aging through an intermediate state. We quantified the evolving landscape for revealing the dynamic characteristics of aging through certain regulation changes in time. We also predicted the key genes and regulations for fast-aging and slow-aging through the analysis of the stability for landscape basins. We also found the oscillation dynamics between fast-aging and slow-aging and showed that more energy is required to sustain such oscillations. We found that the flux is the dynamic cause and the entropy production rate the thermodynamic origin of the phase transitions or the bifurcations between the three-state phase and oscillation phase. The landscape quantification provides a global and physical approach to explore the underlying mechanisms of cellular aging in mammals.

## 1. Introduction

The study of aging has been one of the most long-lasting and influential fields for both scientists and the public. Previous studies have shown that there are nine hallmarks of aging: genomic instability, telomere attrition, epigenetic alterations, loss of proteostasis, deregulated nutrient sensing, mitochondrial dysfunction, cellular senescence, stem cell exhaustion, and altered intercellular communication (López-Otín et al., 2013). In this work, we focus on studies of cellular aging based not only on the key genes but also, more importantly, on their associated gene regulations. Thanks to the rapid development of molecular biology, researchers can manipulate certain genes and observe their effects on the aging process of a model organism (Gems and Partridge, 2013). An early breakthrough showed that the mutation of only one gene, daf-2, can prolong the lifespan of *Caenorhabditis elegans* by more than two times (Kenyon et al., 1993). Since then, hundreds of genes related to aging have been isolated, and evolutionarily conserved pathways like Insulin/IGF-1 signaling, TOR signaling, AMP kinase, and Sirtuins have been identified (Kenyon, 2010; Colman et al., 2014). Although great progress has been made in aging research over the last several decades, there is still a lack of a physical model to integrate these experimental observations, to quantitatively understand the mechanisms of how the internal and external elements (such as environments) work together to control aging, and to predict the key genes and regulations that significantly affect the aging process.

The landscape paradigm for development was introduced by Waddington in the 1940s (Waddington, 2014). However, the Waddington landscape initially only provided a qualitative picture and lacked physical foundation and quantification (Wang, 2015). Recently, there has been significant progress in establishing the physical theory and foundation as well as the quantification of the Waddington landscape (Wang et al., 2008; Wang J. et al., 2011; Wang, 2015; Zhou and Li, 2016). A detailed comparison and critical review of various approaches was presented in (Zhou and Li, 2016). To find the core mechanisms of the mammalian cellular aging process, we built a gene regulatory network based on the existing experimental literature (Haruta et al., 2000; Stambolic et al., 2001; Inoki et al., 2002, 2003; Ogawara et al., 2002; Kong, 2004; Lahav et al., 2004; Nemoto, 2004; You et al., 2006; Greer et al., 2007b; Okoshi et al., 2007; Budanov and Karin, 2008; Gwinn et al., 2008; Lan et al., 2008; Salih and Brunet, 2008; Cantó et al., 2009; Chen et al., 2010; Georgescu, 2010; Ghosh et al., 2010; Sengupta et al., 2010; Yi and Luo, 2010; Budanov, 2011; Dunlop et al., 2011; Gao et al., 2011; Kim et al., 2011; Löffler et al., 2011; Renault et al., 2011; Wang F. et al., 2011; Parmigiani et al., 2014). We quantified the potential landscape through analyzing the long-term dynamic trajectories. We identified the driving forces of aging dynamics as the steady-state probability landscape and the steady-state probability flux. While the landscape tends to stabilize the states of the system, the flux tends to stabilize the flow of state. The quantification of the landscape and the flux provides us with a global way to understand the functions and stabilities of and also the relationships among different functional states. Furthermore, one can detect what key elements can lead to significant changes on the system stabilities and quantify these by the landscape topography through barrier heights and switching times between states.

In the following sections, we first detail how we built an underlying gene regulatory network of mammalian cellular aging based on the existing experimental literature (Haruta et al., 2000; Stambolic et al., 2001; Inoki et al., 2002, 2003; Ogawara et al., 2002; Kong, 2004; Lahav et al., 2004; Nemoto, 2004; You et al., 2006; Greer et al., 2007b; Okoshi et al., 2007; Budanov and Karin, 2008; Gwinn et al., 2008; Lan et al., 2008; Salih and Brunet, 2008; Cantó et al., 2009; Chen et al., 2010; Georgescu, 2010; Ghosh et al., 2010; Sengupta et al., 2010; Yi and Luo, 2010; Budanov, 2011; Dunlop et al., 2011; Gao et al., 2011; Kim et al., 2011; Löffler et al., 2011; Renault et al., 2011; Wang F. et al., 2011; Parmigiani et al., 2014). Based on this gene circuit, we developed a mathematical model to quantitatively describe the basic features of the mammalian cellular aging process. A landscape with three attractors that represent fast-aging, intermediate, and slow-aging, respectively, was identified. We discuss the biological functions of these three attractors and their possible effects on mammalian cellular aging. We identify the dominant paths of system switching between the fast-aging and slow-aging state attractors, giving the most likely route of how fast-aging and slow-aging processes may have occurred. Since the cellular aging process is affected by many factors from inside and outside of the system, we performed a global sensitivity analysis based on the landscape topography and kinetics to investigate how the changes of the genes and the regulations influence the fast-aging and slow-aging processes. The genes or regulations that may play key roles in controlling the mammalian cellular aging process are predicted. Finally, we also found a possible scenario of oscillation dynamics between fast-aging and slow-aging. We show the phase transition/bifurcation between a multi-stable state and oscillation of fast-aging and slow-aging. We show that the flux is the dynamic cause and entropy production rate related to the flux the thermodynamic cause for this phase transition/bifurcation process of fast-aging and slow-aging.

## 2. Results

### 2.1. Network Wiring and Kinetic Equations

**Figure 1:**
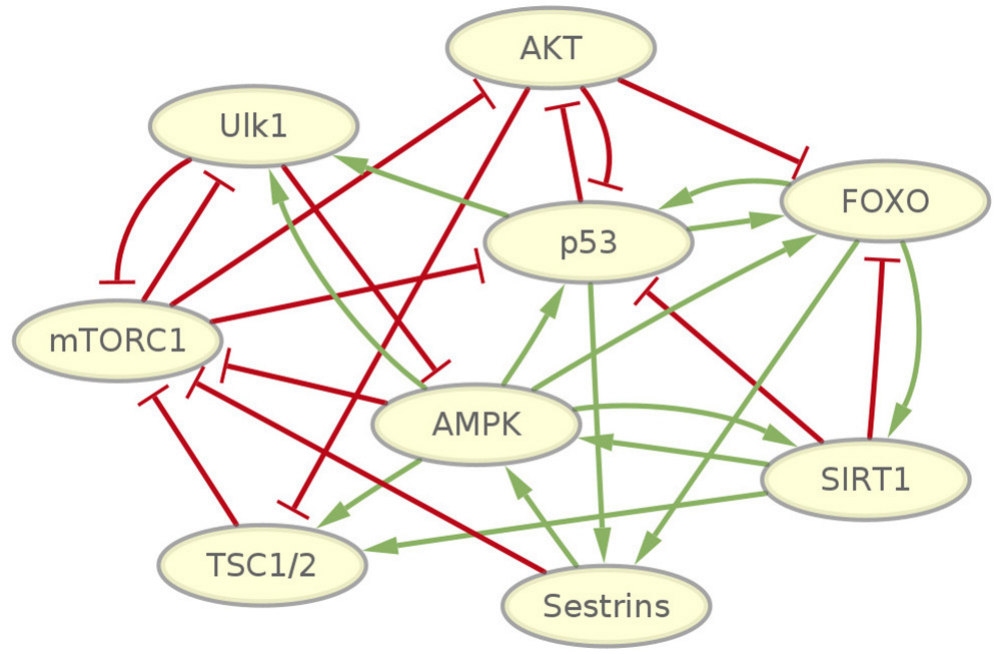
Gene network wiring of mammalian cellular aging. Green arrows represent activation regulations. Red bars represent inhibition regulations.

To investigate the fundamental dynamic features of mammalian cellular aging, we first selected genes that have been revealed to play essential roles in aging. We then gathered the regulatory information regarding these genes by mining the literature for previous relevant studies (Haruta et al., 2000; Stambolic et al., 2001; Inoki et al., 2002, 2003; Ogawara et al., 2002; Kong, 2004; Lahav et al., 2004; Nemoto, 2004; You et al., 2006; Greer et al., 2007b; Okoshi et al., 2007; Budanov and Karin, 2008; Gwinn et al., 2008; Lan et al., 2008; Salih and Brunet, 2008; Cantó et al., 2009; Chen et al., 2010; Georgescu, 2010; Ghosh et al., 2010; Sengupta et al., 2010; Yi and Luo, 2010; Budanov, 2011; Dunlop et al., 2011; Gao et al., 2011; Kim et al., 2011; Löffler et al., 2011; Renault et al., 2011; Wang F. et al., 2011; Parmigiani et al., 2014). We integrated all of this information to give rise to a gene regulatory network. This gene regulatory network of the mammalian cellular aging includes nine genes and 28 regulatory interactions, as shown in Figure 1.

Some well-studied genes and pathways related to mammalian cellular aging are included in the network. The PI3K/Akt signaling pathway, which inhibits FOXO transcription factors, is highly conserved across metazoans (Hay, 2011). FOXO transcription factors have consistently been revealed as important determinants in aging and longevity. In mammals, the FOXO subfamily is involved in a wide range of crucial cellular processes regulating stress resistance, metabolism, cell cycle arrest, and apoptosis (Martins et al., 2015). AMPK and mTORC1 are important nutrient-sensing protein kinases that have antagonistic functions in regulating metabolic homeostasis. Several experiments show that inhibiting mTORC1 delays aging in yeast and invertebrates, extends lifespan in mice, and has an impact on a diverse array of age-related diseases (Johnson et al., 2013). An increase in AMPK activity extends lifespan in lower organisms (Salminen and Kaarniranta, 2012), and experiments demonstrated that AMPK together with mTORC1 and ULK1, a key protein needed in the early steps of autophagosome biogenesis, controls cell growth and autophagy in mammals (Huber et al., 2012; Dunlop and Tee, 2013). Inactivation of Sestrin genes in invertebrates resulted in diverse metabolic pathologies, including oxidative damage, fat accumulation, mitochondrial dysfunction, and muscle degeneration, which resemble accelerated tissue aging (Lee et al., 2013). SIRT1 regulates numerous processes, including inflammation and cellular senescence and aging (Rahman and Bagchi, 2013). SIRT1 is decreased in both transcriptional and post-transcriptional conditions during aging, accompanied by attenuated mitochondrial biogenesis, an important component of aging-related diseases (Yuan et al., 2016). The p53 gene is well-known as a tumor suppressor gene. Its activation also modulates cellular senescence and organismal aging. P53 also regulates aging in a complex way. It accelerates or decelerates the aging process under different circumstances (Rufini et al., 2013). Besides, a few important regulatory interactions affecting aging have been studied. It is found that an AMPK-FOXO pathway is important for mediating life span extension by caloric restriction in *C. elegans* (Greer et al., 2007a). AMPK regulation of FOXO factors may help coordinate energy metabolism with cellular responses to prevent diabetes (Greer et al., 2007b). FOXO3 and p53 are part of a common transcriptional network affecting cellular and organismal responses that is important to counter aging and cancer (Renault et al., 2011). The p53-regulated antioxidant Sestrins gene family involved in control of the AMPK-TORC1 pathway and mitochondrial function might defend against the accumulation of detrimental damage, which potentiates aging and fuel age-associated diseases (Budanov, 2011). It has been found that SIRT2 deacetylates FOXO3 to increase the expression of its target genes, thus regulating cell proliferation, anti-oxidation, and apoptosis (Wang et al., 2007). Detailed references for each regulatory interaction in the network can be found in Table S1.

The complexity of the network wiring is reflected in two different aspects. From the molecular biological perspective, several types of regulatory interactions are present in the network, including transcriptional regulation, translational control, protein-protein interaction, and signal transduction. From the network wiring topology perspective, the intensive communications among the nine genes imply emergent biological functions as a result. The network motif includes positive and negative, feed-forward and feed-back loops. This can give rise to the possibility of generating complex dynamic features, such as forming multi-stable state attractors and oscillations.

To explore the dynamics of the mammalian cellular aging network, we employ non-linear differential equations (Tyson and Novák, 2010) to describe the dynamics of each genes expression in the network. A sigmoidal function was previously used to model T-cell differentiation (Hong et al., 2012) and epithelial-mesenchymal transition (Watanabe et al., 2019) in mammalian cells and appears to be suitable for describing both gene expression and gene regulation networks (Mjolsness et al., 1991; Hong et al., 2011, 2015). There are nine genes in the network, so a total of nine equations are included in our simulation model. The form of the kinetic equation is shown as:

(1) $$\begin{array}{l} \dot{X_{i}}=\gamma_{i}\left[F\left(\sigma_{i}W_{i}\right)-X_{i}\right] \end{array}$$

(2) $$\begin{array}{l} F\left(\sigma_{i}W_{i}\right)=1/\left(1+e^{-\sigma_{i}W_{i}}\right) \end{array}$$

(3) $$\begin{array}{l} W_{i}=\omega_{i0}+\underset{j}{\sum }\omega_{ij}X_{j} \end{array}$$

where *X*_*i*_ represents the expression level of the gene i, where i = 1,.,9, in the network. The parameter γ_*i*_ denotes a reciprocal rate description of the dynamic timescale of the system. *F*(σ_*i*_*W*_*i*_) denotes the regulation for gene i. It is described by a non-linear sigmoidal function that varies from 0 where *W*_*i*_ ≪ −1/σ_*i*_ to 1 where *W*_*i*_ ≫ 1/σ_*i*_. *W*_*i*_ denotes a combination of the effects of all input regulations to gene i. A small regulation input to gene i will lead to a weak driving force for the dynamics of gene i, while a large regulation input to gene i will give rise to a large driving force to the dynamics of gene i. The coefficient ω_*ij*_ indicates the regulatory strength of gene j on gene i, where ω_*ij*_ < 0 for inhibitory interaction, ω_*ij*_ > 0 for promoting regulation, and ω_*ij*_ = 0 for no effect of gene j on gene i. The coefficient ω_*i*0_ represents the basal regulation strength. Since cellular aging is not an isolated or static process, the value of ω_*i*0_ can be varied under genetic changes or environmental influences. The parameter σ_*i*_ controls the steepness of the sigmoidal function at its inflection point. It provides a threshold for the onset of significant dynamics of the gene.

### 2.2. Potential Landscape of Aging

A biological system is naturally subject to intrinsic and extrinsic fluctuations. Therefore, we added an additional fluctuation term in the ODEs to characterize the stochastic behaviors of the mammalian cellular aging process. We use the Langevin dynamic approach to simulate the gene circuit dynamics. From the resulting dynamic trajectories of the gene expressions, we collected the statistics and quantified the underlying potential landscape (Wang J. et al., 2011). For visualization, we projected the high-dimensional state spaces into two coordinates. This choice can still distinguish the major biological functions that are reflected as attractors in the landscape.

**Figure 2:**
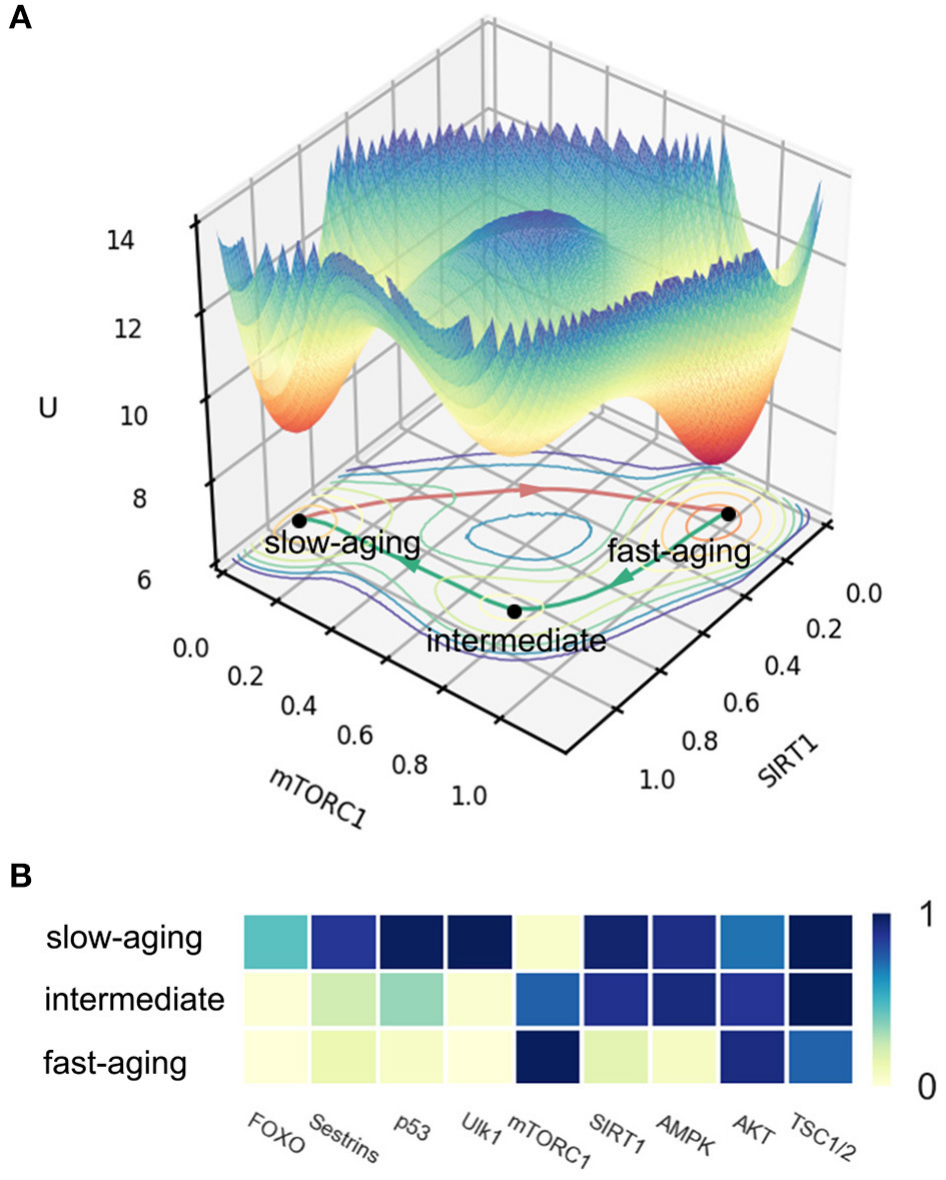
The potential landscape and gene expression levels of fast-aging and slow-aging. Red arrows represent the dominant path from slow-aging to fast-aging. Green arrows represent the dominant paths from fast-aging to slow-aging. **(A)** The potential landscape of fast-aging and slow-aging. **(B)** Gene expression levels of fast-aging and slow-aging.

The potential landscape of mammalian cellular aging is shown in Figure 2A. *X* and *Y* coordinates represent the expression levels of genes SIRT1 and mTORC1, respectively. The *Z* coordinate represents the landscape *U*. Three attractors emerge on the 3D landscape. The position of each attractor can be distinguished by the expression levels of all of the nine genes in the network. This is shown as a heatmap in Figure 2B. We defined the three attractors as the slow-aging state (S), fast-aging state (F), and intermediate state (I) according to the gene expression levels and their corresponding gene functions. In the slow-aging state, genes with longevity-promoting functions, such as SIRT1, AMPK, and Ulk1, have relatively high expression levels and genes with lifespan-limiting effects, such as mTORC1 and AKT, have relatively low expression levels. In the fast-aging state, longevity-promoting genes and lifespan-limiting genes show the opposite expression patterns compared to the case of slow aging. The intermediate state is located between the fast-aging and the slow-aging state; genes, such as FOXO, Sestrins, p53, and Ulk1 show relatively low expression levels compared to the slow-aging state, while genes, such as SIRT1 and AMPK show relatively high expression levels compared to the slow-aging state. Some organisms undergo rapid aging and death, while others grow old slowly and live far longer, even within a population of isogenic organisms in identical environments (Crane et al., 2020). A previous study on an aging model of yeast cell with an intermediate state was proposed based on categorizing the age-dependent phenotypic conditions and was validated through experiment (Jin et al., 2019). The emergence of the intermediate state provides new perspectives to explain the mechanisms of the mammalian cellular aging process. The intermediate state may provide a bridge or mid-land between fast-aging and slow-aging. This can help to facilitate the fast or slow aging process through the intermediate state.

The depths of the three attractors are significantly different. A deeper attractor has lower energy *U*, where *U* = −*logP* and *P* represents the steady-state probability of the state. Thus, the system is expected to reside in a deeper attractor for a longer time, and it is harder to escape from it. The mean first passage time (MFPT) reflects the average transition time from one attractor to another. In Figure 2A, we can see the fast-aging state attractor is deeper than the slow-aging state attractor and the intermediate attractor. We calculate the MFPT from slow-aging to fast-aging and from fast-aging to slow-aging as 44.27 and 126.32, respectively. These quantifications indicate that under current system conditions, the system prefers to stay at the fast-aging attractor with lifespan-limiting effects, and the transition from slow-aging to fast-aging is significantly faster than that from fast-aging to slow-aging. This may explain why the fast-aging process seems more dominant, since the fast-aging state attractor is more stable and therefore has a higher chance of being observed.

The dominant path (Wang et al., 2010) is the most probable path when a system switches from one state to another. We quantify the dominant paths from slow-aging to fast-aging and from fast-aging to slow-aging, which are separately shown as a red arrow and a green arrow in Figure 2A. It is notable that the two dominant paths are completely different. For the fast-aging process, the red dominant path is directly from the slow-aging state to fast-aging state. The green dominant path from fast-aging to slow-aging passes through the intermediate state. This indicates that, in our mammalian cellular aging model, the slow-aging process is divided into two steps. The first step is from the fast-aging state to the intermediate state, marked by a significant increase in the expression levels of AMPK and the SIRT1. These two genes together regulate diverse processes, such as cellular fuel metabolism, inflammation, and mitochondrial function (Ruderman et al., 2010). The second step is from the intermediate state to the slow-aging state, marked by the changes of the gene expressions of the other aging-related genes, such as FOXO and mTORC1 (see Figure 2B). It is possible to experimentally slow the rate of aging through longevity genes or dietary restriction (Rando and Chang, 2012), but further experimental verifications are needed to check the predictions of the two-step transition to slow-aging in our model.

### 2.3. Dynamics of Landscapes of Aging

Aging is certainly not an isolated process. Most of the aging-related genes are multi-faced, and they also play key roles in some other basic functions, such as metabolism, energy homeostasis, protein synthesis, cell growth, proliferation, autophagy, apoptosis, and senescence. Several kinds of stimulations have been found to have a great influence on the natural aging process. Genetic manipulations of certain genes have been found to significantly extend the lifespan of *C. elegans* (McCormick et al., 2011). Dietary restrictions have been found to regulate aging and increase the healthy lifespan in various model organisms (Kapahi et al., 2010; Smith-Vikos et al., 2014). The accumulation of cell damage was shown to lead to several types of degenerative diseases like cancer and Alzheimers disease (Powers et al., 2009). These examples reflect the importance of studying aging in a systematic and dynamic way.

In our mammalian cellular aging model, the parameter ω_*i*0_ in the ODEs represents the basal expression level for each gene i. The increase or decrease of ω_*i*0_ will influence the behaviors of the system. The dynamic landscapes describe the changes in the landscape topography according to the changes in certain genes or regulations. The barrier height (*BH*) based on principal component analysis (PCA) of the landscape can be used to quantitatively measure the degree of difficulty for the system to switch from one attractor to another. *BH* is defined as the difference between the minimum potential in the current attractor and the potential of the saddle point from the current attractor to the other attractor. We first use the PCA method to project the nine-dimensional landscape into the top two principal components (PCs). We then calculate the BH among the attractors based on the PCA projected landscape.

**Figure 3:**
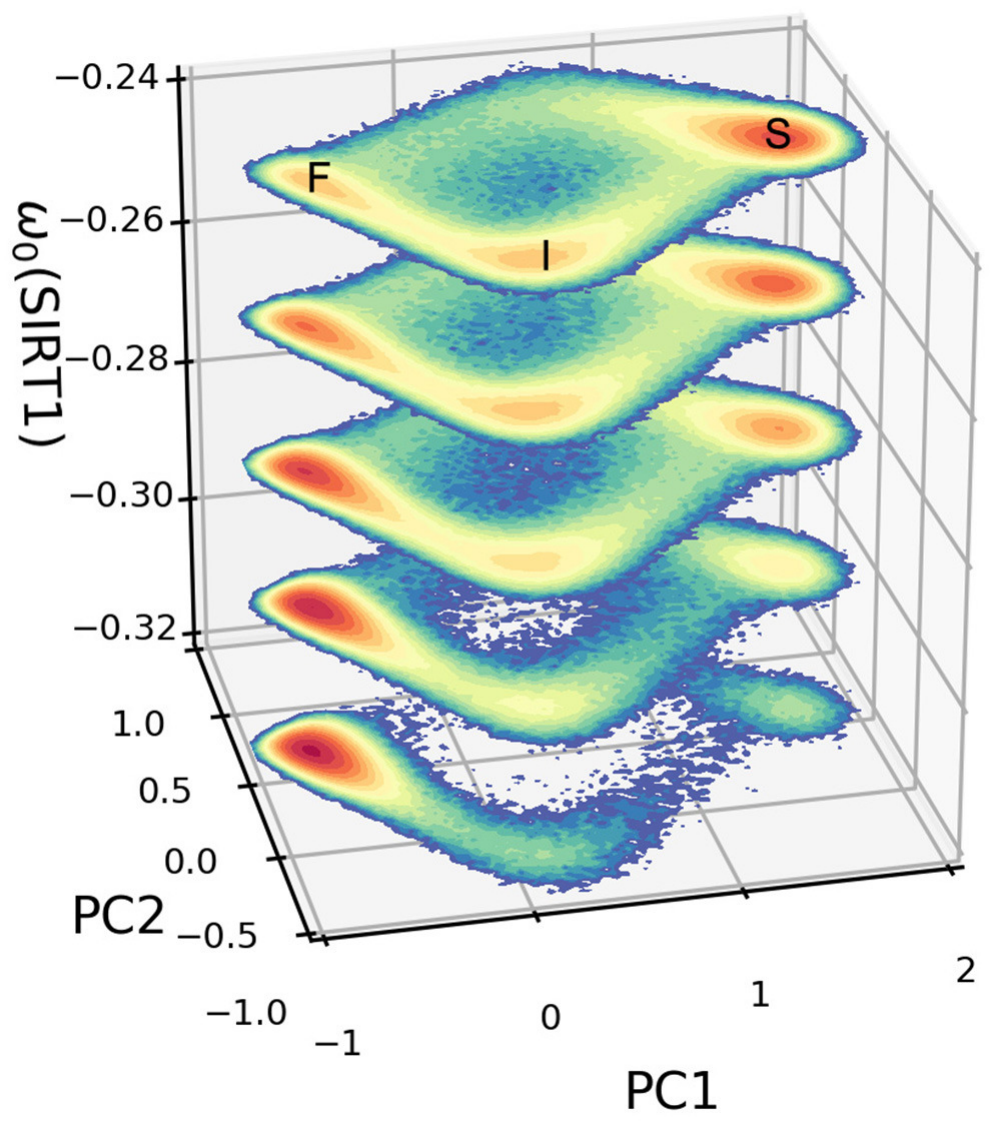
Dynamic landscape of fast-aging and slow-aging upon changes in the basal expression level of SIRT1. The horizontal coordinates represent the top two principal components of gene expression, while the vertical axis represents changes in the basal level of expression for gene SIRT1.

**Figure 4:**
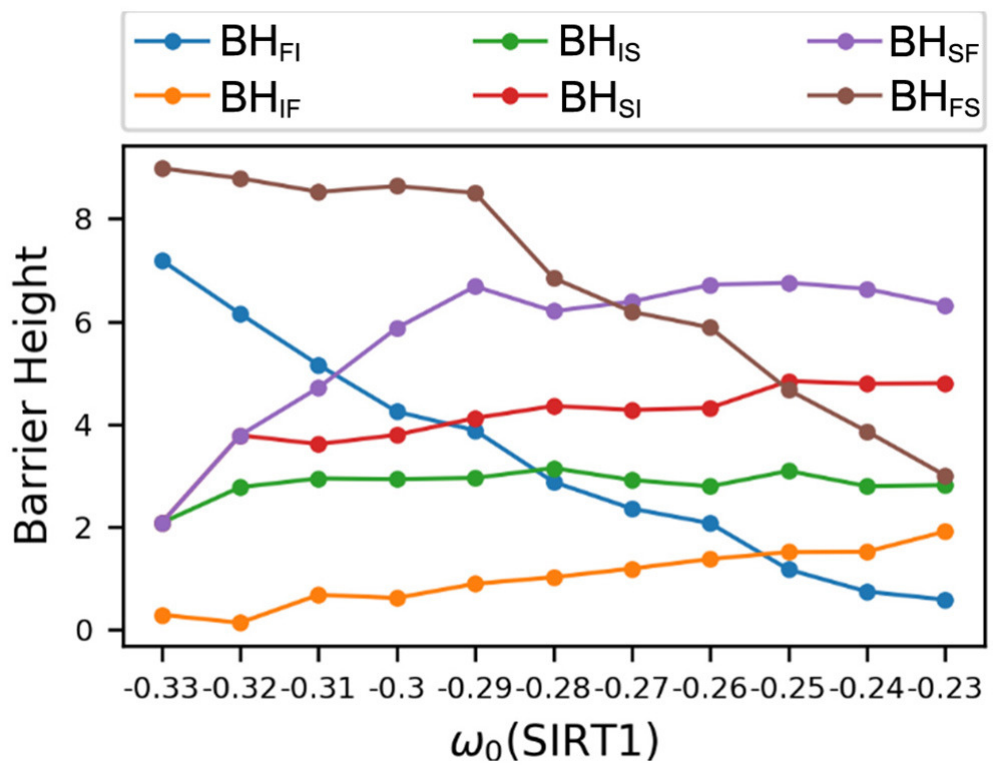
Changes in barrier heights upon increasing the basal gene expression level of SIRT1. *BH*_*FI*_, barrier height from the fast-aging to the intermediate attractor; *BH*_*IF*_, barrier height from the intermediate to the fast-aging attractor; *BH*_*IS*_, barrier height from the intermediate to the slow-aging attractor; *BH*_*SI*_, barrier height from the slow-aging to the intermediate attractor; *BH*_*SF*_, barrier height from the slow-aging to the fast-aging attractor; *BH*_*FS*_, barrier height from the fast-aging to the slow-aging attractor.

The dynamical PCA landscapes according to the changes in the basal expression level of SIRT1 are shown in Figure 3. The *X* and *Y* coordinates represent the top two principal components, respectively. These two principal components show about 95 percent of the variance of the dynamic expression trajectory. The three attractors are labeled F (fast-aging), I (intermediate), and S (slow-aging), respectively. If we increase (decrease) the SIRT1 basal expression level, the depth of the fast-aging attractor decreases (increases) and the depths of both the intermediate and slow-aging attractors increase (decrease). Figure 4 quantitatively shows the change of barrier height vs. the increase in basal expression level of SIRT1. There are three attractors in the PCA landscape, so a total of six barrier heights for each pair of attractors can be quantified. The line labeled *BH*_*SF*_ denotes the BH of the system switching from the slow-aging to the fast-aging attractor, while *BH*_*FS*_ represents the BH of the system switching from the fast-aging to the slow-aging attractor. Other labels have similar notations. The results clearly show an increase (decrease) in the stability of the fast-aging attractor state and a decrease (increase) in the stability for the slow-aging and intermediate states when the SIRT1 basal expression level decreases (increases). These results are consistent with the evidence that SIRT1 plays a key role in dietary restriction-induced longevity promotion, while the activity of SIRT1 decreases with the mammalian cellular aging process (Ruderman et al., 2010).

### 2.4. Global Sensitivity Analysis of Aging in Mammals

Here, we use global sensitivity analysis to quantitatively identify the contributions of individual regulations on the functional behavior of mammalian cellular aging. We change the basal expression level ω_*i*0_ for every gene and the regulatory strength for every regulation ω_*ij*_ to investigate to what extent these regulations influence the functional behavior. The functional stability can be quantitatively measured by the barrier heights. *BH*_0_ represents the barrier height with the original value of the given parameter. Δ*BH* represents changes in the barrier height when the regulation is changed by a constant value (0.04). Thus, Δ*BH*/*BH*_0_ can be used to measure the sensitivity of the barrier heights under certain regulation changes. We performed global sensitivity analysis to find the key genes or regulations by changing ω_*i*0_ or ω_*ij*_ and then finding out which genes and regulations will significantly impact the landscape stability. These predicted genes or regulations may play important roles in the mammalian cellular aging process or may even be useful in treating aging-related degenerative diseases.

**Figure 5:**
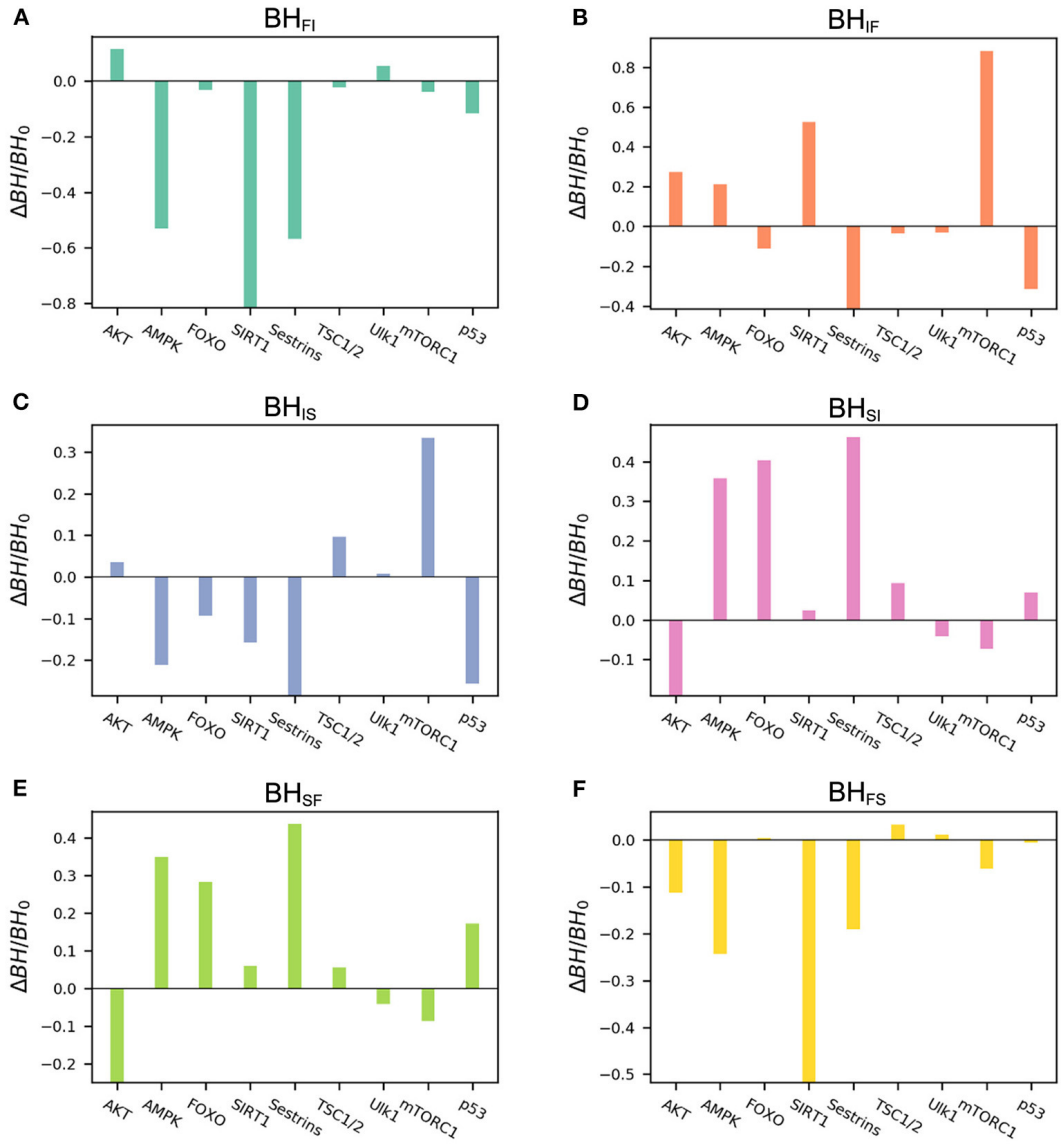
Results of global sensitivity analysis of the barrier height upon changing the basal expression level of different genes. *BH*_0_, barrier height with the original value; Δ*BH*, change in barrier height upon changing the basal expression level. **(A)** Change in barrier height from the fast-aging to the intermediate attractor. **(B)** Change in barrier height from the intermediate to the fast-aging attractor. **(C)** Change in barrier height from the intermediate to the slow-aging attractor. **(D)** Change in barrier height from the slow-aging to the intermediate attractor. **(E)** Change in barrier height from the slow-aging to the fast-aging attractor. **(F)** Change in barrier height from the fast-aging to the slow-aging attractor.

We performed global sensitivity analysis on the basal expression level to quantifying the barrier height changes for every gene. The detailed results of the global sensitivity analysis are shown in Figure 5. For the barriers related to the slow-aging state, *BH*_*SF*_ and *BH*_*SI*_, we can see that increasing the basal expression levels of the genes AMPK, FOXO, and Sestrins significantly enhances the stability of the slow-aging state. This indicates that it becomes harder for the system to escape from the slow-aging state. In contrast, gene AKT significantly decreases the stability of the slow-aging state. These results are consistent with previous experimental findings (Salminen and Kaarniranta, 2012; Lee et al., 2013; Gharibi et al., 2014; Martins et al., 2015). For the barrier heights related to the fast-aging state, *BH*_*FI*_ and *BH*_*FS*_, we can clearly see that increasing the basal expression levels of the genes AMPK, SIRT1, and Sestrins significantly decreases the stability of the fast-aging state. AMPK and Sestrins play opposite roles in the slow-aging state, but the role of SIRT1 in slow-aging is not significant. For the intermediate state, the result is complex. Genes mTORC1 and p53 are only effective in the intermediate state, but not in the other two states. Although the existence of the intermediate state between fast-aging and slow-aging has not been directly verified, this study shows that different genes seem to influence different attractors. This can provide new insight for research on mammalian cellular aging mechanisms.

**Figure 6:**
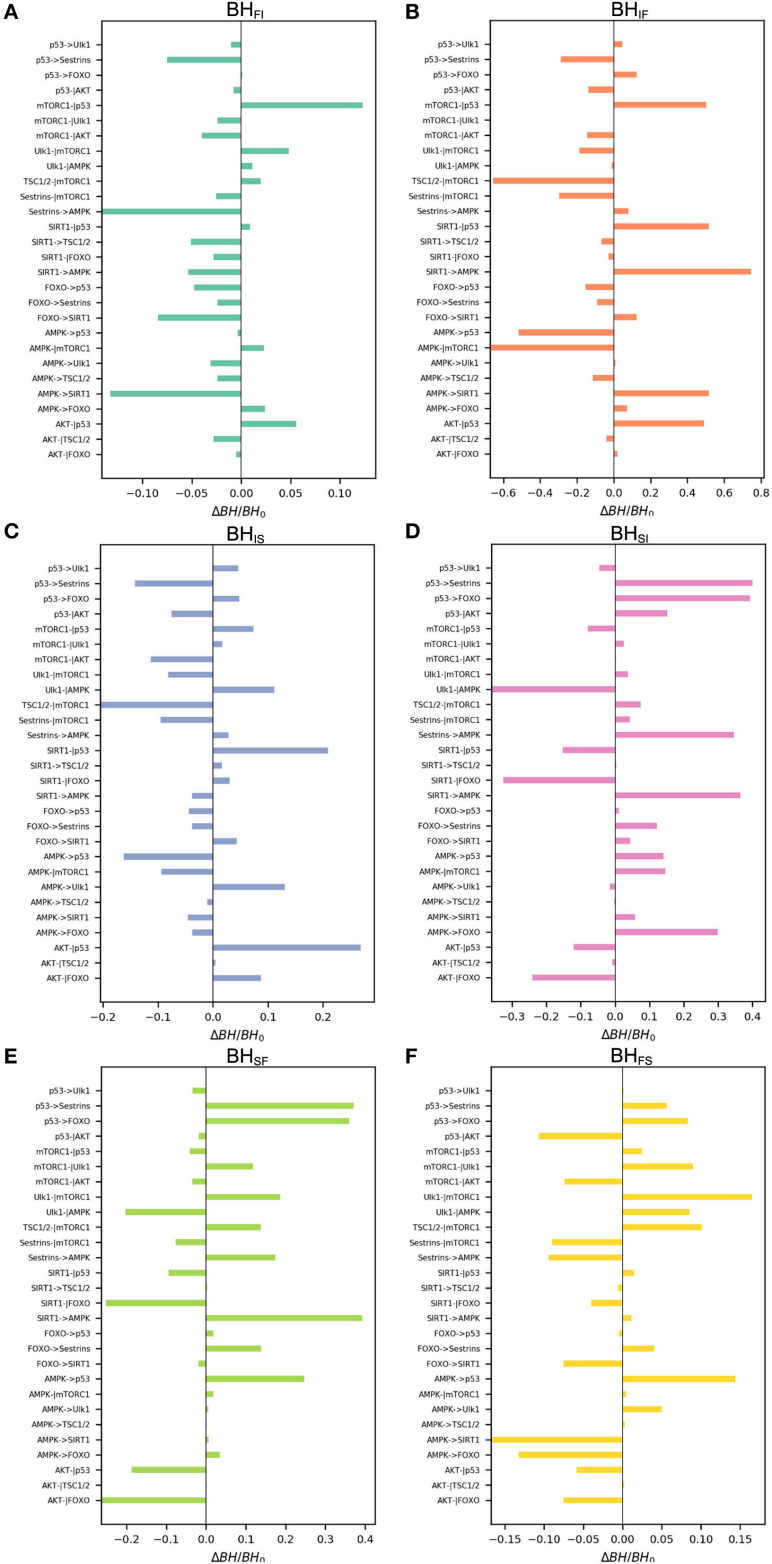
Global sensitivity analysis of the barrier height upon changing regulatory strengths. *BH*_0_, barrier height with the original value; Δ*BH*, change in barrier height upon changing the regulation. **(A)** Change in barrier height from the fast-aging to the intermediate attractor. **(B)** Change in barrier height from the intermediate to the fast-aging attractor. **(C)** Change in barrier height from the intermediate to the slow-aging attractor. **(D)** Change in barrier height from the slow-aging to the intermediate attractor. **(E)** Change in barrier height from the slow-aging to the fast-aging attractor. **(F)** Change in barrier height from the fast-aging to the slow-aging attractor.

We also performed global sensitivity analysis on regulatory strength ω_*ij*_. The bar charts shown in Figure 6 reflect Δ*BH* = *BH*_0_ vs. ω_*ij*_. The most sensitive regulation from the slow-aging state to the fast-aging state is SIRT1->AMPK, and the barrier height from the slow-aging state to the fast-aging state is increased with increasing SIRT1->AMPK. This means that increasing the activation regulation of SIRT1->AMPK will stabilize the slow-aging state and therefore delay the aging process. The most sensitive regulation of barrier height from the fast-aging state to the slow-aging state is AMPK->SIRT1, and the barrier height from the fast-aging state to slow-aging state is decreased with increasing AMPK->SIRT1. This means that increasing the activation regulation of AMPK->SIRT1 will destabilize the fast-aging state and therefore increase the chance of slow aging, thereby delaying the aging process. The most sensitive regulation of barrier height from the intermediate state to the slow-aging state is AKT-|p53, and the barrier height from the intermediate state to the slow-aging state is increased with increasing AKT-|p53. This means that increasing the inhibition regulation of AKT-|p53 will stabilize the intermediate state and decrease the chance of slow aging, effectively promoting the aging process. The most sensitive regulation of barrier height from the slow-aging state to the intermediate state is p53->Sestrins, and the barrier height from the slow-aging state to the intermediate state is increased with increasing p53->Sestrins. This means that increasing the activation regulation of p53->Sestrins will stabilize the slow-aging state and therefore delay the aging process. The most sensitive regulation of barrier height from the fast-aging state to the intermediate state is Sestrins->AMPK, and the barrier height from the fast-aging state to the intermediate state is decreased with increasing Sestrins->AMPK. This means the increasing the activation regulation of Sestrins->AMPK will destabilize the fast-aging state and therefore increase the chance of slow aging, thus effectively delaying the aging process. The most sensitive regulation of barrier height from the intermediate state to the fast-aging state is SIRT1->AMPK, and the barrier height from the intermediate state to the fast-aging state is decreased with increasing SIRT1->AMPK. This means that increasing the activation regulation of SIRT1->AMPK will stabilize the intermediate state and destabilize the fast-aging state and therefore delay the aging process. We show the top three sensitive regulations for each barrier in Table 1. Changes in these regulatory strengths significantly change the system behavior. Further experiments are needed to validate these predictions.

**Table 1:** Top three key regulations from global sensitivity analysis.

**Barrier**	**Sensitive regulations**
*BH*_*SF*_	SIRT1->AMPK, p53->Sestrins, p53->FOXO.
*BH*_*FS*_	AMPK->SIRT1, Ulk1-|mTORC1, AMPK->p53.
*BH*_*IS*_	AKT-|p53, SIRT1-|p53, TSC1/2-|mTORC1.
*BH*_*SI*_	p53->Sestrins, p53->FOXO, SIRT1->AMPK.
*BH*_*FI*_	Sestrins->AMPK, AMPK->SIRT1, mTORC1-|p53.
*BH*_*IF*_	SIRT1->AMPK, AMPK-|mTORC1, TSC1/2-|mTORC1.

### 2.5. Aging Oscillations Landscape

**Figure 7:**
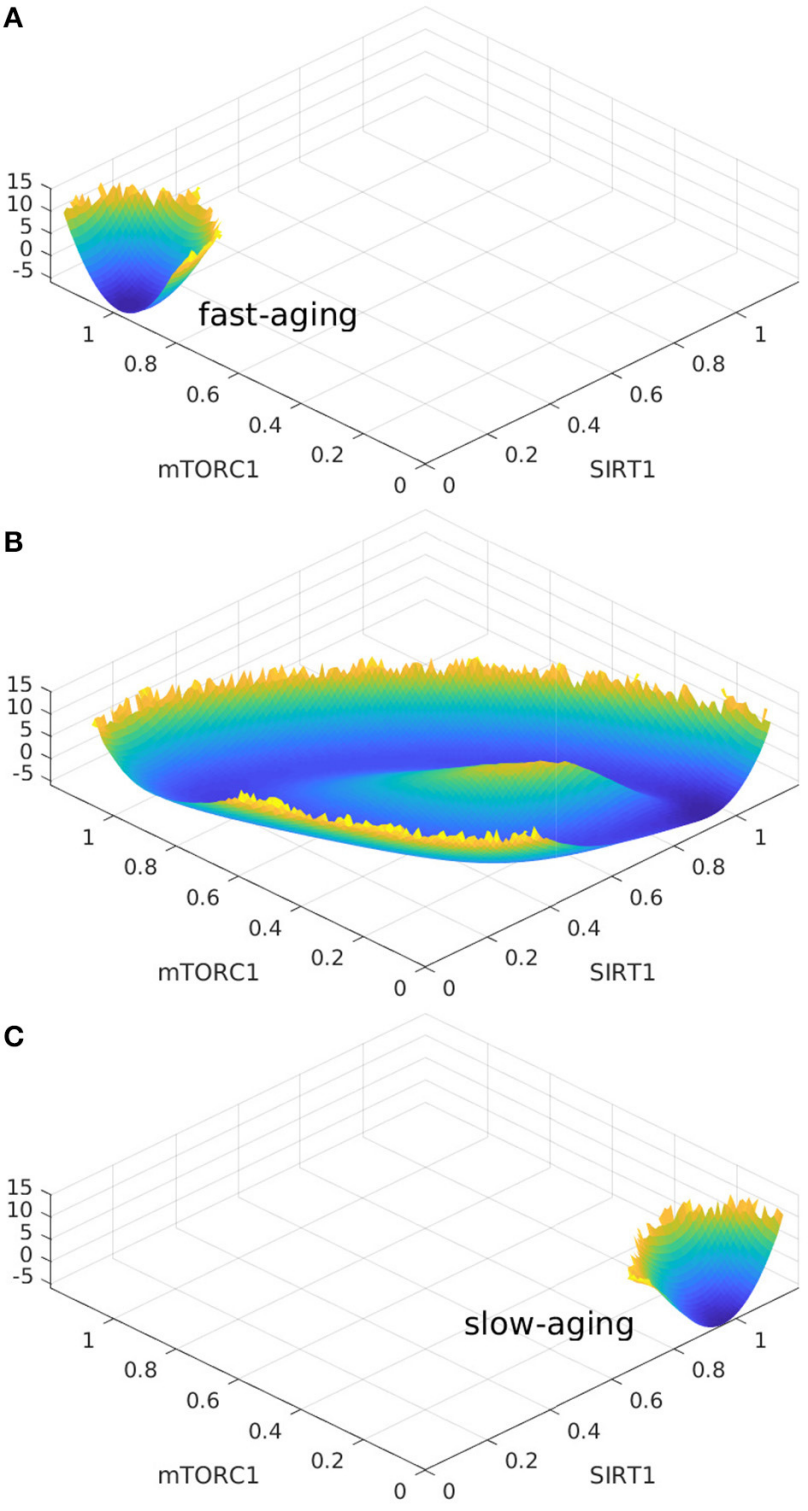
The landscape topography changes from the monostable state of fast-aging to the oscillation between the fast-aging and the slow-aging, and then to the monostable slow-aging state upon the increase of the regulation of Sestrins->AMPK. **(A)** The landscape of fast-aging. **(B)** The landscape of oscillation between fast-aging and slow-aging. **(C)** The landscape of slow-aging.

Oscillation dynamics can emerge in certain parameter regimes when the regulation strengths are varied. The transitions between the oscillation and monostable states are found to be mainly regulated by Sestrins->AMPK. The changes in landscape topography are shown in Figure 7. RS represents the regulation strength of Sestrins->AMPK. The landscape shows oscillation dynamics with a Mexican hat shape when RS is 0.76, as shown in Figure 7B. The two relatively deeper regions on the oscillation ring correspond to the fast-aging and slow-aging state, respectively. The states of the system rotate clockwise along the oscillation ring valley around the central hill of the Mexican hat. When the regulation strength RS is increased, the slow-aging state attractor becomes deeper. When the regulation strength RS is increased to 0.88, the system switches from the oscillation to a monostable state with only the slow-aging attractor state. In contrast, when the regulation strength RS is decreased, the basin at the fast-aging steady state becomes deeper. When the regulation strength RS is decreased to 0.62, the system switches from the oscillation to a monostable state with only the fast-aging steady state.

**Figure 8:**
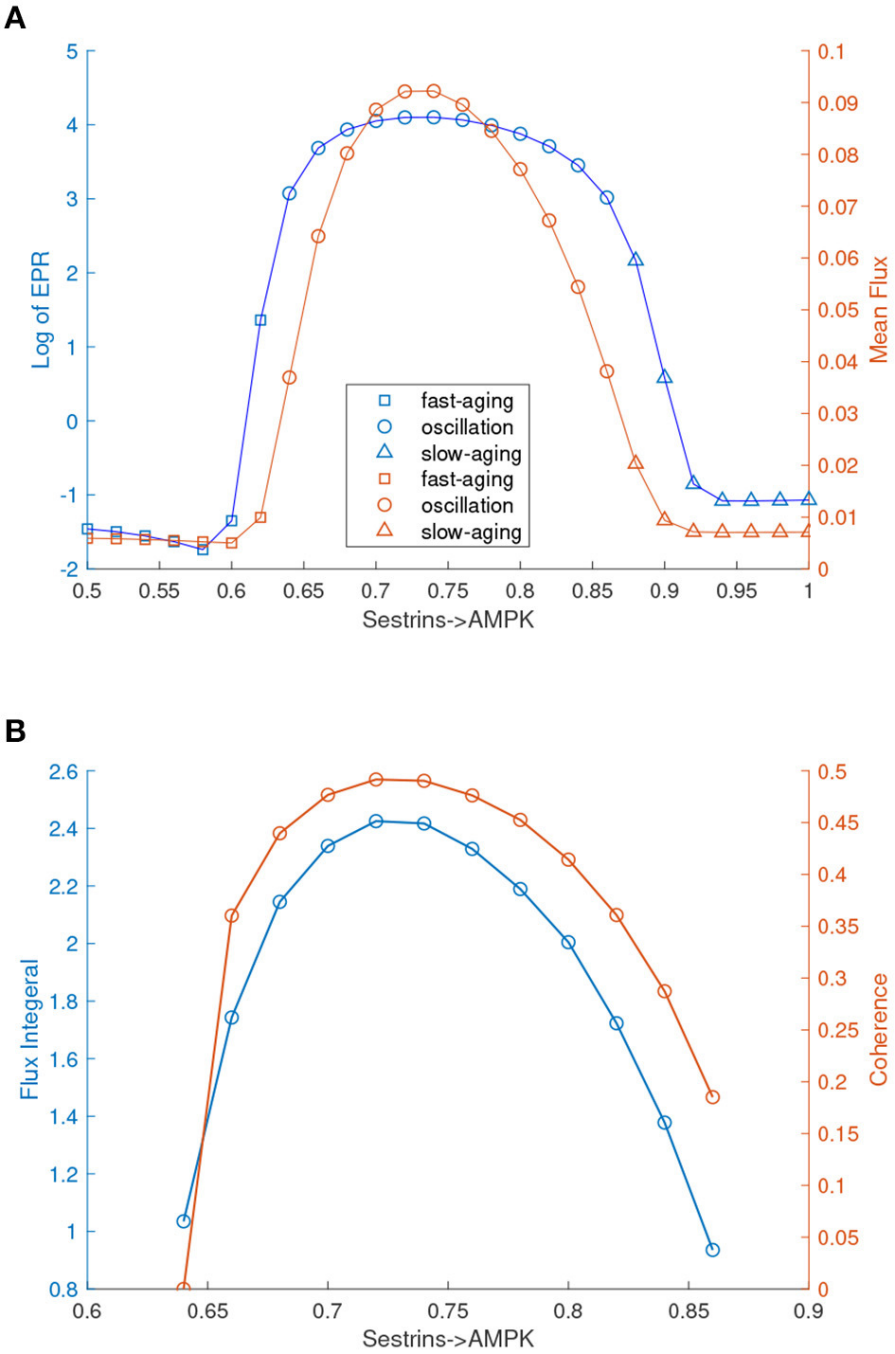
EPR, flux, and coherence changes upon the regulation changes of Sestrins->AMPK through the transitions from the monostable fast-aging state to the oscillation between the fast-aging state and the slow-aging state, and then to the slow-aging state. **(A)** The entropy production rate and the mean flux of the monostability and the oscillation. **(B)** The flux integral and the coherence of oscillations.

Interestingly, these oscillation dynamics were found in the previous mathematical model of *C. elegans* (Zhao and Wang, 2016). The oscillation can drive the dynamics to switch coherently (periodically) between the fast-aging state and the slow-aging state. The processes of fast-aging and slow-aging occur at different times along with the oscillation. The transitions between the fast-aging state and the slow-aging state with the oscillation are different from the transitions in a tri-stable system. The transitions between the fast-aging state and the slow-aging state in the tri-stable regime are random and incoherent, while the transitions between the fast-aging state and the slow-aging state in the oscillation regime are periodic and coherent. In order to address the role of the flux as the driving force of the aging process in addition to the landscape, we quantified the flux integral as a measure of the magnitude of the flux and the coherence of the oscillation, as shown in Figure 8B. The flux integral correlates with the coherence. This indicates that higher flux leads to more stable and coherent oscillation.

We also quantified the thermodynamic cost in terms of the entropy production rate (EPR), which is related to the flux and the mean flux, for the phase transition/bifurcation from the monostability of fast-aging to oscillation and from the oscillation to monostability of slow-aging by increasing the regulation strength of Sestrins->AMPK. An increase in the EPR indicates that the system costs more energy to maintain. The mean flux correlates with the EPR. As shown in Figure 8A, the EPR is low when the system stays in the phase of the fast-aging state. When the strength of Sestrins->AMPK increases, the EPR increases sharply at the phase where the transition from the stable fast-aging state to oscillation occurs. When the system switches from oscillation to the monostable slow-aging state, the EPR sharply decreases and then stays at a low level. This demonstrates that the oscillation costs more energy to maintain than either the fast-aging or slow-aging state. Through the oscillation, the dynamic process of switching between fast-aging and slow-aging achieves functional switching, which can cost more energy. Therefore, there can be direct and indirect pathways for aging. The direct pathway is the one directly from the slow-aging state to the fast-aging state. The indirect pathways can be from the slow-aging state to the fast-aging state through either the intermediate state or oscillation.

## 3. Discussion

In this study, we presented a mathematical model to describe the dynamic features of the mammalian cellular aging process. We built the underlying gene regulatory network by integrating the information from previous experimental studies. The genes and wirings in the gene regulatory network were formed, and the dynamics of gene expression was described by nine non-linear ordinary differential equations. Based on these equations, we quantified the potential landscape of the mammalian cellular aging process. Three attractors emerged on the landscape: the fast-aging, intermediate, and slow-aging states. When the system resides in one of the three attractors, the escape time is determined by the depth of the attractor. The system can also switch from one attractor to another, and the transition needs to overcome the barriers between the attractors. We integrated the previous studies and analyzed the mammalian cellular aging process from a systemic and network perspective.

The aging process is not only a spontaneous biological process but also can be significantly altered by interventions, such as genetic manipulations and dietary restrictions. Thus, the potential landscape of aging is not invariant. We changed certain regulations in our model in order to perform quantitative analysis and investigate the changes in aging functions through the changes in the landscape. The stabilities of attractors can be significantly changed by the basal strength of certain genes and the regulatory strengths of gene-gene regulations. We believe that these genes or regulations may play key roles in the mammalian cellular aging process. Further experiments are needed to validate these predictions.

Oscillations emerge in certain regulation regimes. The oscillation leads to switching between the processes of fast-aging and slow-aging. This is different from switching between the fast-aging state and the slow-aging state through the stochastic trajectories in the tri-stable regime. The switching between the fast-aging state and the slow-aging state in the oscillation regime is periodic and coherent. In contrast, the switching between the fast-aging state and the slow-aging state in the tri-stable regime is random and incoherent. Through the analysis of the flux integral and coherence as well as the mean flux and entropy production rate, it is suggested that more energy is required to sustain oscillations.

In this work, we have provided a framework to reveal the underlying mechanism of fast-aging and slow-aging in mammals based on landscape and flux theory. We predict the key genes and interactions in the fast-aging and slow-aging processes. This approach may be helpful for studying strategies for expanding lifespan in mammals or humans.

## 4. Materials and Methods

### 4.1. Kinetic Equations

**Figure 9:**
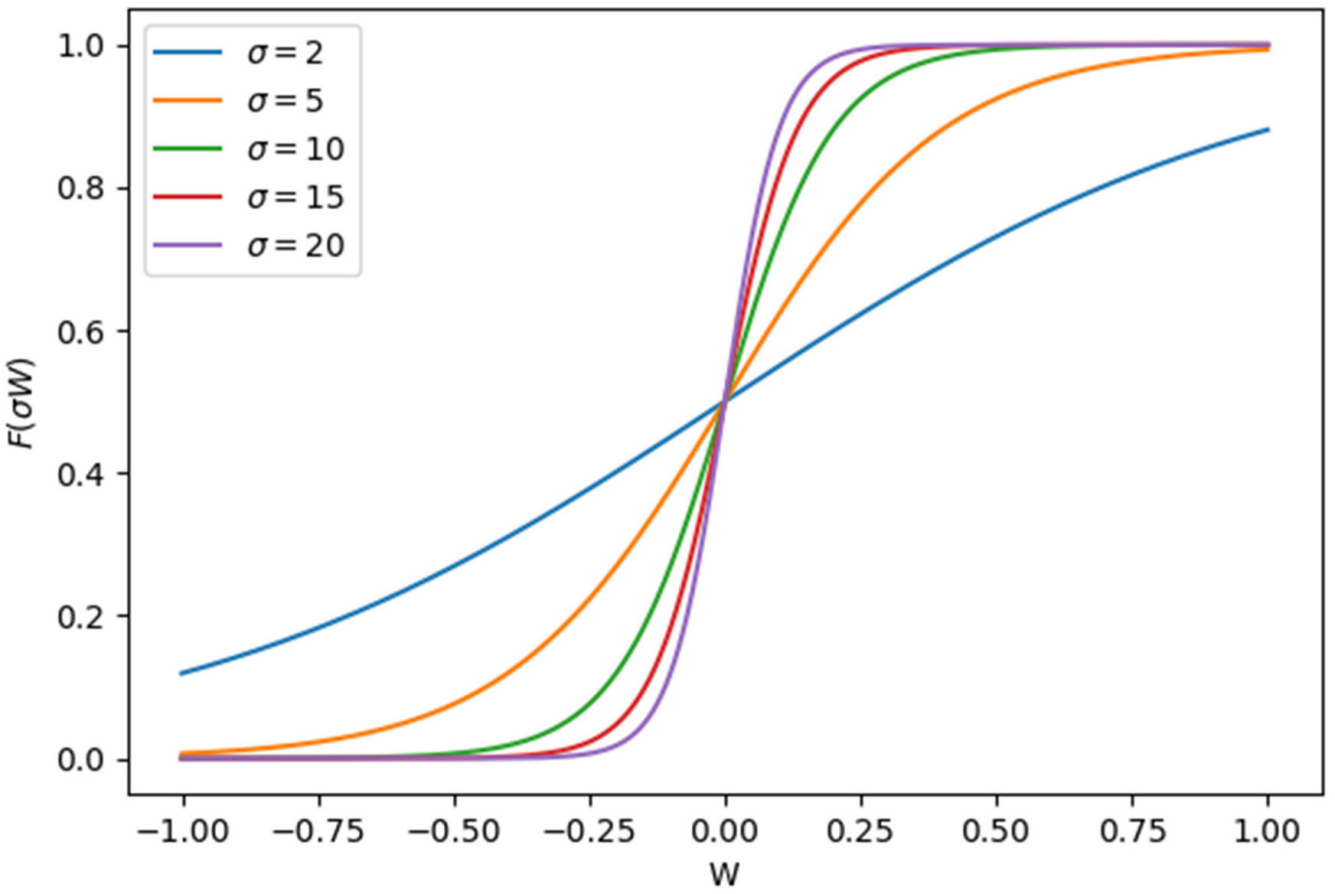
Shape of the force function *F*(σ*W*) vs. different values of *W*. *W*, the combination of effects of all input regulation on a certain gene.

The shape of the term *F*(σ*W*) in the non-linear ODEs used in our model is intuitively similar to Hill equations. The sigmoidal shape and the steepness of *F*(σ*W*) can be altered by varying certain parameters, as shown in Figure 9.

The form of summing Hill equations as the regulation force for ODEs is used in other studies (Li, 2018; Li and Balazsi, 2018). It is shown in Equation (4).

(4) $$\begin{array}{l} \frac{dx_{k}}{dt}=\underset{i\in activators}{\sum }\frac{w_{k}x_{i}^{n}}{s_{ik}^{n}+x_{i}^{n}}+\underset{j\in inhibitors}{\sum }\frac{w_{k}s_{jk}^{n}}{s_{jk}^{n}+x_{j}^{n}}-\mu_{k}x_{k} \end{array}$$

where *x*_*k*_ represents the kth gene expression, while *w*_*k*_ represents the relative strength of every regulation of gene k. Parameter μ_*k*_ is defined as the self-degradation rate. Parameter n is the Hill coefficient. Parameters *s*_*ik*_ and *s*_*jk*_ represent the inflection points of the activation or inhibition regulation terms.

However, the Hill equations have an inherent defect that the value of *w* cannot be negative. This leads to defects in the case of the presence of both activation and inhibition regulations. For example, under the additive rule, when adding a negative regulation or increasing the weight of a negative regulation, the expression changes, and *dx*/*dt* may increase, while in fact it should decrease. This problem also emerges when using the multiplicative rule.

In the equations in our model, the regulations of activation and inhibition have the same form, ω_*ij*_*X*_*j*_, as shown in Equations (1–3). The coefficient ω_*ij*_ indicates the regulatory strength from gene j to gene i, where ω_*ij*_ < 0 for inhibitory interaction and ω_*ij*_ > 0 for promoting regulation. The value of *Ẋ*_*i*_ is increased when *W*_*i*_ is increased. *W*_*i*_ is increased or decreased when an activation term or inhibition term is added. Thus, *Ẋ*_*i*_ is increased when an activation term is added and is decreased when an inhibition term is added. This overcomes the defects of the form representing the regulation in Equation (4). Therefore, we can directly add the regulation term, ω_*ij*_*X*_*j*_, together to quantify the force. The calculation is logically reasonable and less time-consuming.

### 4.2. Parameter Setting

We assume the following restrictions on the regulation parameters. For all equations, the steepness parameter is set to σ = 10, and the time scale parameter is set to γ = 1. The basal weight parameter is set to −1 < ω_*i*0_ < 1, and the regulation weight parameter is set to −1 < ω_*ij*_ < 1. This equation has the great advantage that it is subject to all the powerful analytical and simulation tools of non-linear ODEs. This is because in the limit of large σ_*i*_, it behaves like a discrete Boolean network. When σ ≫ 1, *X*_*i*_ tends to flip between 0 and 1, and the dynamic system describes a Boolean network.

There is a question about why and how to set the basal weight parameter ω_*i*0_. Technically, ω_*i*0_ has to be present because if only activation or inhibition on a target node X exists, the expression level of X will eventually reach the boundary values, 1 and 0. This can make it hard for multi-stability to emerge. Biologically, the model we developed can be influenced by the environment, and many conditions and molecular signals from outside can change the state of the system and affect the basal level of the expressions. The parameter settings of ω_*ij*_ and ω_*i*0_ are shown in Table S2.

### 4.3. Langevin Method

The aging process in real life is influenced by the intrinsic or external fluctuations of the system. Langevin equation is appropriate to describe the stochastic time evolution of gene expression dynamics. These stochastic differential equations for describing to gene regulatory network dynamics are as follows:

(5) $$\begin{array}{l} \dot{\text{x}}=\text{F}\left(\text{x}\right)+\eta \end{array}$$

where **x** is a vector of the gene expressions and **F**(**x**) is the driving force of the gene-regulating network dynamics. The term **η** represents fluctuation or noise force, which has a Gaussian probability distribution with correlation function $<\eta_{i}\left(t\right),\eta_{j}\left(t^{\prime }\right)>=2D_{ij}\delta_{ij}\delta \left(t-t^{\prime }\right)$, where **D** is the diffusion coefficient matrix characterizing the strength of the fluctuations. The global steady-state probability distribution *P* for the state space can be quantified through the statistics by collecting the time evolution trajectories of the expression dynamics from long-duration simulations.

### 4.4. Landscape and Flux

The individual stochastic trajectory is unpredictable due to its random nature. However, the evolution of the probability distribution is predictable and can be used to describe the probabilistic behaviors and patterns of the aging process. The evolution of the probability distribution is governed by the Fokker-Planck equation (Wang et al., 2008; Wang, 2015) as follows:

(6) $$\begin{array}{l} \frac{\partial P\left(\text{x},t\right)}{\partial t}=-\nabla \cdot \text{J}\left(\text{x},t\right) \end{array}$$

(7) $$\begin{array}{l} \text{J}\left(\text{x},t\right)=\text{F}\left(\text{x}\right)P\left(\text{x},t\right)-\text{D}\cdot \nabla P\left(\text{x},t\right) \end{array}$$

which presents that the change in the probability *P*(**x**, *t*) in time at state **x** and time t is equal to the probability flux **J**(**x**, *t*) in or out of this state at time t characterized by its divergence. In the steady state, the divergence of probability flux is equal to zero. However, the probability flux is not necessarily equal to zero. The steady-state probability flux, due to its divergent free nature, is rotational as a curl. The steady-state probability flux at the steady state (long time limit) is given in Equation (7). The steady-state probability flux being not equal to zero represents net flow to or from the system. The non-zero net flow breaks the detailed balance. Therefore, the steady-state probability flux quantifies the degree of non-equilibrium away from the equilibrium when it has deviated from zero. For non-equilibrium systems, the driving force F for the dynamics can be decomposed to a gradient of the potential landscape and a curl flux force under constant fluctuations (Wang et al., 2008): **F** = −**D** · ∇*U* + **J**_**ss**_/*P*_*ss*_, where *U* = −*lnP*_*ss*_ is the potential landscape, while Pss is the steady-state probability distribution.

### 4.5. Dominant Path

The dominant paths are the most probable paths when the system switches from one state to another. The quantification of the dominant paths is important for uncovering how the biological processes have actually occurred and is therefore the key for understanding the underlying physical mechanism and function. The dominant path can be quantified by the path integral approach (Wang et al., 2010; Wang J. et al., 2011). The probability of switching from the initial **x** at time 0 to the final **x** at time t with the path integral is given as:

(8) $$\begin{array}{l} P\left({\text{x}}_{\text{final}},t,{\text{x}}_{\text{initial}},0\right)=\int D\text{x}exp[-\int dt(\frac{1}{2}\nabla \cdot \text{F}\left(\text{x}\right)\\ \;+\frac{1}{4}\left(\dot{\text{x}}-\text{F}\left(\text{x}\right)\right))\cdot {\text{D}}^{-1}\cdot \left(\dot{\text{x}}-\text{F}\left(\text{x}\right)\right)]\\ \;=\int D\text{x}exp\left[-S\left(\text{x}\right)\right]\\ \;=\int D\text{x}exp\left[-\int L\left(\text{x}\left(t\right)\right)dt\right] \end{array}$$

The integral over *D***x** represents the sum over all the possible trajectories from the state **x**_**initial**_ at time 0 to the state **x**_**final**_ at time t. **F**(**x**) represents the driving force of the gene regulatory network dynamics. **D** represents the strength of the diffusion coefficient matrix. *S*(**x**) and *L*(**x**(*t*)) represent the action and the Lagrangian of the associated path. Each path is assigned with a probability weight, *exp*[−*S*(**x**)], associated with the action of that path. The dominant path is the path with the largest weight. Therefore, the dominant paths can be identified through minimizing the action.

### 4.6. Entropy Production Rate

A non-equilibrium system often exchanges energy, matter, and information with the environment. This leads to thermodynamic dissipation. The change in the system entropy in the non-equilibrium system can be divided into two parts (Wang et al., 2008; Wang, 2015) as:

(9) $$\begin{array}{l} Ṡ=\dot{S_{t}}-\dot{S_{e}} \end{array}$$

(10) $$\begin{array}{l} \dot{S_{t}}=\int dx\left(\text{J}\cdot {\text{D}}^{-1}\cdot \text{J}\right)/P \end{array}$$

(11) $$\begin{array}{l} \dot{S_{e}}=\int dx\left(\text{J}\cdot {\text{D}}^{-1}\right)\cdot {\text{F}}^{\prime } \end{array}$$

where $\dot{S_{t}}$ represents the entropy production rate (EPR) or the total entropy rate of the system and environment and $\dot{S_{e}}$ represents the heat dissipation rate of the environments. The effective force **F**′ is defined as **F**′ = **F** − ∇ · **D**.

## Data Availability Statement

All datasets generated for this study are included in the article/Supplementary Material.

## Author Contributions

JW designed the research. WL, LZ, and JW performed the research, analyzed the data, and wrote the paper. All authors contributed to the article and approved the submitted version.

## Conflict of Interest

The authors declare that the research was conducted in the absence of any commercial or financial relationships that could be construed as a potential conflict of interest.
