# Corrigendum: Searching for the Mechanisms of Mammalian Cellular Aging Through Underlying Gene Regulatory Networks

**DOI:** 10.3389/fgene.2021.684415

**Published:** 2021-05-20

**Authors:** Wenbo Li, Lei Zhao, Jin Wang

**Affiliations:** ^1^State Key Laboratory of Electroanalytical Chemistry, Changchun Institute of Applied Chemistry, Chinese Academy of Sciences, Changchun, China; ^2^Department of Chemistry and Physics, State University of New York at Stony Brook, Stony Brook, NY, United States

**Keywords:** aging, slow-aging, landscape, flux, entropy production, gene regulatory network

In the published article, there are various errors due to a mistake in the labels in Figure 2B. The labels for fast-aging and slow-aging are incorrect and should be exchanged. The corrected Figure 2 appears below.

**Figure 2:**
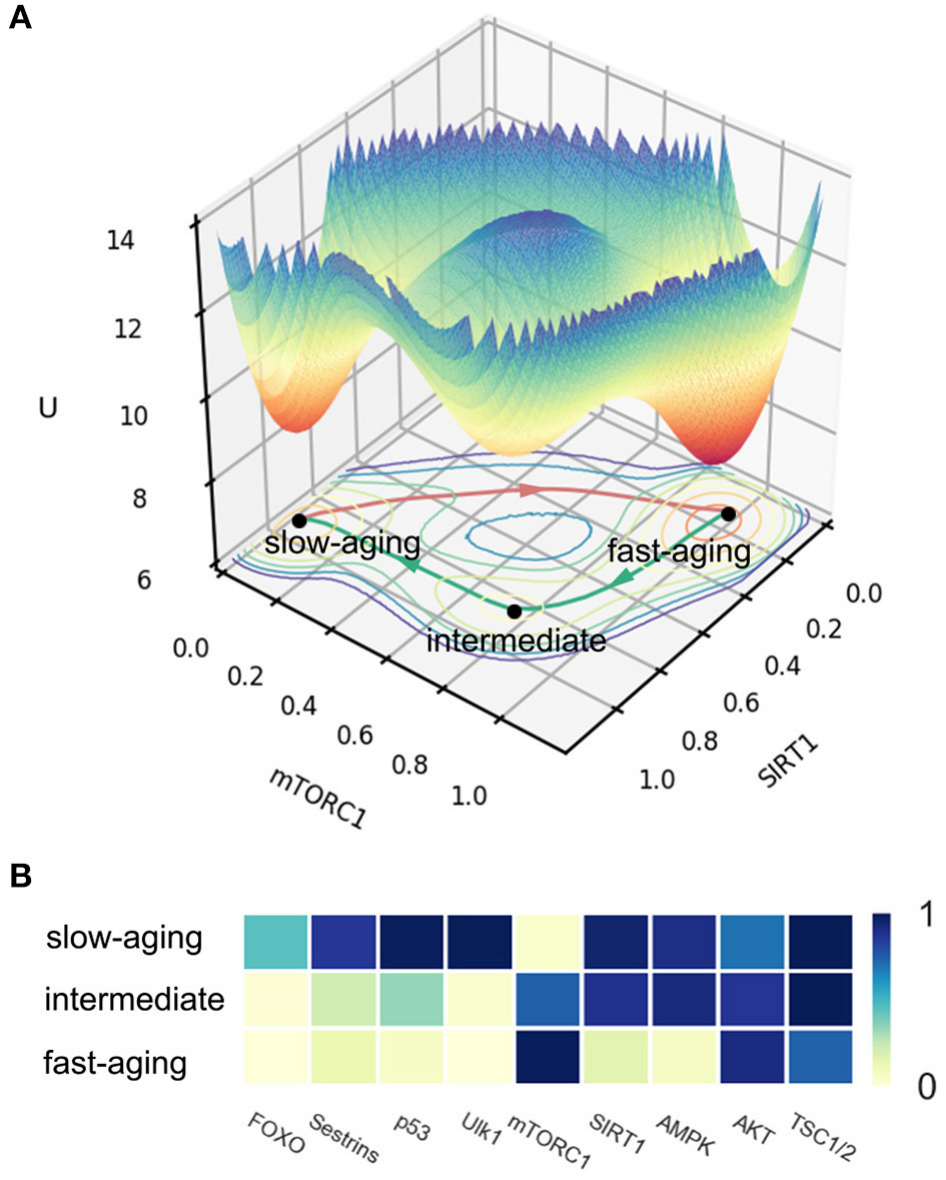
The potential landscape and gene expression levels of fast-aging and slow-aging. Red arrows represent the dominant path from slow-aging to fast-aging. Green arrows represent the dominant paths from fast-aging to slow-aging. **(A)** The potential landscape of fast-aging and slow-aging. **(B)** Gene expression levels of fast-aging and slow-aging.

Consequently, Figure 7A and Figure 7C should be exchanged. The corrected Figure 7 appears below.

**Figure 7:**
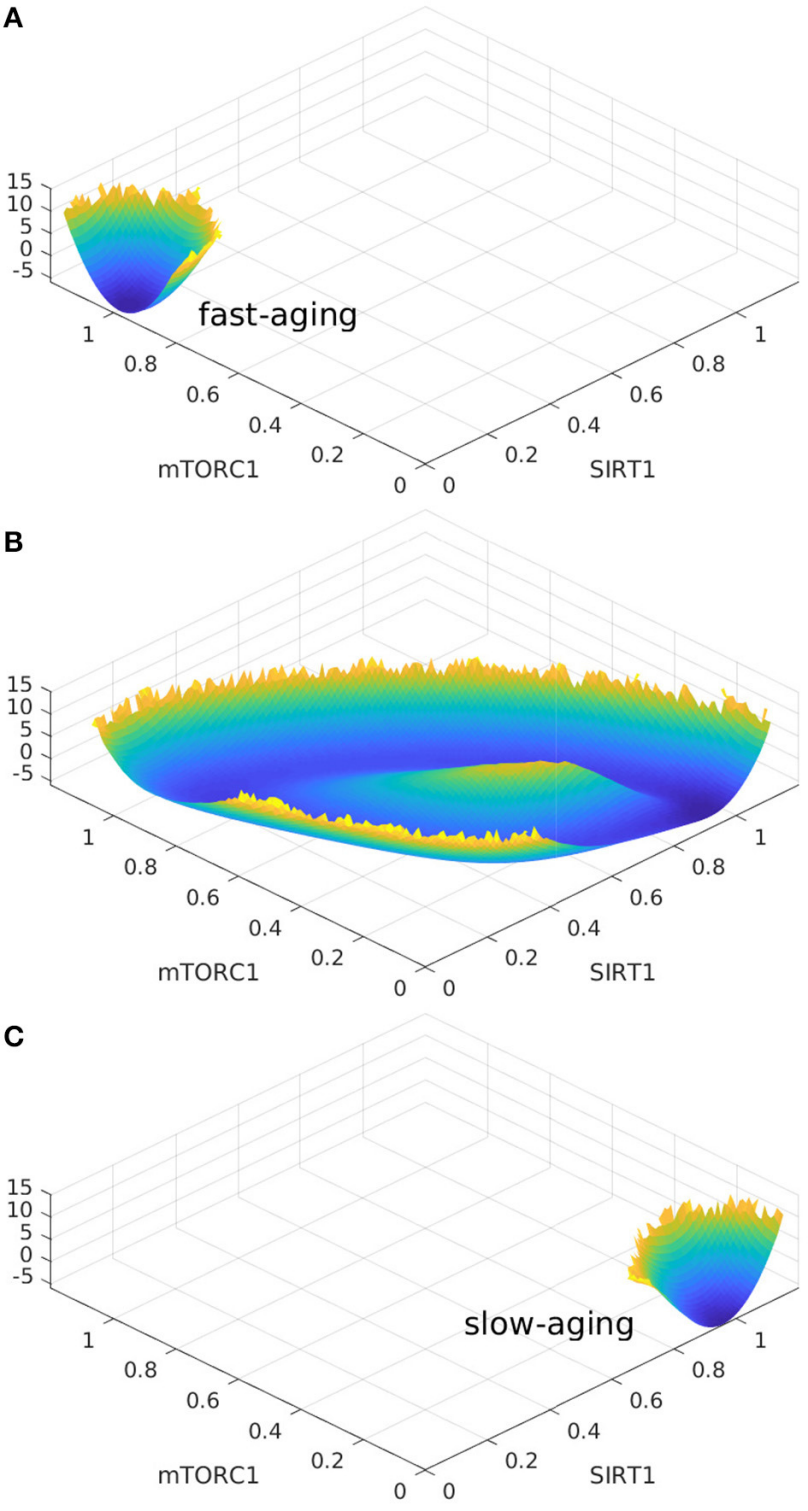
The landscape topography changes from the monostable state of fast-aging to the oscillation between the fast-aging and the slow-aging, and then to the monostable slow-aging state upon the increase of the regulation of Sestrins->AMPK. **(A)** The landscape of fast-aging. **(B)** The landscape of oscillation between fast-aging and slow-aging. **(C)** The landscape of slow-aging.

In Figure 8A, the labels for fast-aging and slow-aging are incorrect and should be exchanged. The corrected Figure 8 appears below.

**Figure 8:**
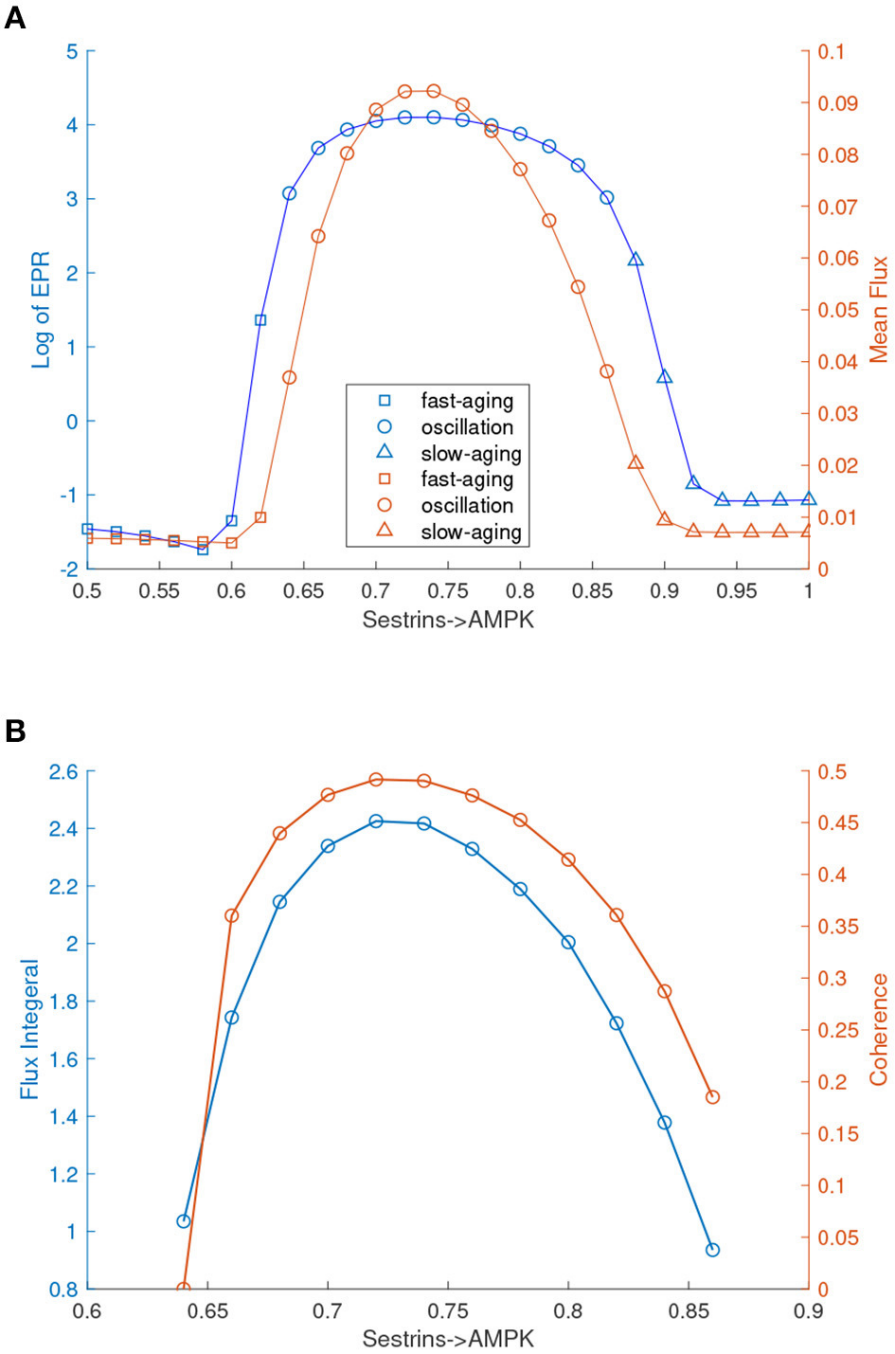
EPR, flux, and coherence changes upon the regulation changes of Sestrins->AMPK through the transitions from the monostable fast-aging state to the oscillation between the fast-aging state and the slow-aging state, and then to the slow-aging state. **(A)** The entropy production rate and the mean flux of the monostability and the oscillation. **(B)** The flux integral and the coherence of oscillations.

Also, there are spelling errors in 2.4. Global Sensitivity Analysis of Aging in Mammals, Paragraph 2. *BH*_*FS*_ should be changed to *BH*_*SF*_ and *BH*_*IS*_ should be changed to *BH*_*SI*_. *BH*_*IF*_ should be changed to *BH*_*FI*_ and *BH*_*SF*_ should be changed to *BH*_*FS*_.

Corrections have been made to 2.4. Global Sensitivity Analysis of Aging in Mammals, Paragraph 2:

We performed global sensitivity analysis on the basal expression level to quantifying the barrier height changes for every gene. The detailed results of the global sensitivity analysis are shown in Figure 5. For the barriers related to the slow-aging state, **BH**_***SF***_ and **BH**_***SI***_, we can see that increasing the basal expression levels of the genes AMPK, FOXO, and Sestrins significantly enhances the stability of the slow-aging state. This indicates that it becomes harder for the system to escape from the slow-aging state. In contrast, gene AKT significantly decreases the stability of the slow-aging state. These results are consistent with previous experimental findings (Salminen and Kaarniranta, 2012; Lee et al., 2013; Gharibi et al., 2014; Martins et al., 2015). For the barrier heights related to the fast-aging state, **BH**_***FI***_ and **BH**_***FS***_, we can clearly see that increasing the basal expression levels of the genes AMPK, SIRT1, and Sestrins significantly decreases the stability of the fast-aging state. AMPK and Sestrins play opposite roles in the slow-aging state, but the role of SIRT1 in slow-aging is not significant. For the intermediate state, the result is complex. Genes mTORC1 and p53 are only effective in the intermediate state, but not in the other two states. Although the existence of the intermediate state between fast-aging and slow-aging has not been directly verified, this study shows that different genes seem to influence different attractors. This can provide new insight for research on mammalian cellular aging mechanisms.

The mistake in 2.4. Global Sensitivity Analysis of Aging in Mammals, Paragraph 3 is caused by the label mistake in Figure 2B. The word decreased, destabilize, stabilize and promote at the bottom of the paragraph should be corrected.

A correction has been made to 2.4. Global Sensitivity Analysis of Aging in Mammals, Paragraph 3:

We also performed global sensitivity analysis on regulatory strength ω_*ij*_. The bar charts shown in Figure 6 reflect Δ*BH* = *BH*_0_ vs. ω_*ij*_. The most sensitive regulation from the slow-aging state to the fast-aging state is SIRT1->AMPK, and the barrier height from the slow-aging state to the fast-aging state is increased with increasing SIRT1->AMPK. This means that increasing the activation regulation of SIRT1->AMPK will stabilize the slow-aging state and therefore delay the aging process. The most sensitive regulation of barrier height from the fast-aging state to the slow-aging state is AMPK->SIRT1, and the barrier height from the fast-aging state to slow-aging state is decreased with increasing AMPK->SIRT1. This means that increasing the activation regulation of AMPK->SIRT1 will destabilize the fast-aging state and therefore increase the chance of slow aging, thereby delaying the aging process. The most sensitive regulation of barrier height from the intermediate state to the slow-aging state is AKT-|p53, and the barrier height from the intermediate state to the slow-aging state is increased with increasing AKT-|p53. This means that increasing the inhibition regulation of AKT-|p53 will stabilize the intermediate state and decrease the chance of slow aging, effectively promoting the aging process. The most sensitive regulation of barrier height from the slow-aging state to the intermediate state is p53->Sestrins, and the barrier height from the slow-aging state to the intermediate state is increased with increasing p53->Sestrins. This means that increasing the activation regulation of p53->Sestrins will stabilize the slow-aging state and therefore delay the aging process. The most sensitive regulation of barrier height from the fast-aging state to the intermediate state is Sestrins->AMPK, and the barrier height from the fast-aging state to the intermediate state is decreased with increasing Sestrins->AMPK. This means the increasing the activation regulation of Sestrins->AMPK will destabilize the fast-aging state and therefore increase the chance of slow aging, thus effectively delaying the aging process. **The most sensitive regulation of barrier height from the intermediate state to the fast-aging state is SIRT1->AMPK, and the barrier height from the intermediate state to the fast-aging state is decreased with increasing SIRT1->AMPK. This means that increasing the activation regulation of SIRT1->AMPK will stabilize the intermediate state and destabilize the fast-aging state and therefore delay the aging process**. We show the top three sensitive regulations for each barrier in Table 1. Changes in these regulatory strengths significantly change the system behavior. Further experiments are needed to validate these predictions.

These mistakes are caused by the label mistake in Figure 2B. Some statements about oscillation in Figures 7 and 8 should be corrected. Some words of slow-aging should be changed to fast-aging and some words of fast-aging should be changed to slow-aging.

Corrections have been made to 2.5. Aging Oscillations Landscape, Paragraph 1 and 3:

Oscillation dynamics can emerge in certain parameter regimes when the regulation strengths are varied. The transitions between the oscillation and monostable states are found to be mainly regulated by Sestrins->AMPK. The changes in landscape topography are shown in Figure 7. RS represents the regulation strength of Sestrins->AMPK. The landscape shows oscillation dynamics with a Mexican hat shape when RS is 0.76, as shown in Figure 7B. The two relatively deeper regions on the oscillation ring correspond to the fast-aging and slow-aging state, respectively. The states of the system rotate clockwise along the oscillation ring valley around the central hill of the Mexican hat. **When the regulation strength RS is increased, the slow-aging state attractor becomes deeper. When the regulation strength RS is increased to 0.88, the system switches from the oscillation to a monostable state with only the slow-aging attractor state. In contrast, when the regulation strength RS is decreased, the basin at the fast-aging steady state becomes deeper. When the regulation strength RS is decreased to 0.62, the system switches from the oscillation to a monostable state with only the fast-aging steady state**.

**We also quantified the thermodynamic cost in terms of the entropy production rate (EPR), which is related to the flux and the mean flux, for the phase transition/bifurcation from the monostability of fast-aging to oscillation and from the oscillation to monostability of slow-aging by increasing the regulation strength of Sestrins->AMPK**. An increase in the EPR indicates that the system costs more energy to maintain. The mean flux correlates with the EPR. As shown in Figure 8A, the EPR is low when the system stays in the phase of the fast-aging state. When the strength of Sestrins->AMPK increases, the EPR increases sharply at the phase where the transition from the stable fast-aging state to oscillation occurs. When the system switches from oscillation to the monostable slow-aging state, the EPR sharply decreases and then stays at a low level. This demonstrates that the oscillation costs more energy to maintain than either the fast-aging or slow-aging state. Through the oscillation, the dynamic process of switching between fast-aging and slow-aging achieves functional switching, which can cost more energy. Therefore, there can be direct and indirect pathways for aging. The direct pathway is the one directly from the slow-aging state to the fast-aging state. The indirect pathways can be from the slow-aging state to the fast-aging state through either the intermediate state or oscillation.

The authors apologize for these errors and state that this does not change the scientific conclusions of the article in any way. The original article has been updated.

## References

[B1] Gharibi B. Farzadi S. Ghuman M. Hughes F. J. (2014). Inhibition of akt/mTOR attenuates age-related changes in mesenchymal stem cells. Stem Cells. 32, 2256–2266. 10.1002/stem.1709 24659476

[B2] Lee J. H. Budanov A. V. Karin M. (2013). Sestrins orchestrate cellular metabolism to attenuate aging. Cell Metab. 18, 792–801. 10.1016/j.cmet.2013.08.018 24055102 PMC3858445

[B3] Martins R. Lithgow G. J. Link W. (2015). Long live FOXO: unraveling the role of FOXO proteins in aging and longevity. Aging Cell. 15, 196–207. 10.1111/acel.12427 26643314 PMC4783344

[B4] Salminen A. Kaarniranta K. (2012). AMP-activated protein kinase (AMPK) controls the aging process via an integrated signaling network. Ageing Res. Rev. 11, 230–241. 10.1016/j.arr.2011.12.005 22186033

